# Epsilon-near-zero regime for ultrafast opto-spintronics

**DOI:** 10.1038/s44306-024-00025-4

**Published:** 2024-06-03

**Authors:** C. S. Davies, A. Kirilyuk

**Affiliations:** 1 FELIX Laboratory, Radboud University, Nijmegen, The Netherlands; 2 Radboud University, Institute for Molecules and Materials, Nijmegen, The Netherlands

**Keywords:** Ultrafast photonics, Slow light, Nonlinear optics, Magnetic properties and materials, Ferroelectrics and multiferroics, Spintronics

## Abstract

Over the last two decades, breakthrough works in the field of non-linear phononics have revealed that high-frequency lattice vibrations, when driven to high amplitude by mid- to far-infrared optical pulses, can bolster the light-matter interaction and thereby lend control over a variety of spontaneous orderings. This approach fundamentally relies on the resonant excitation of infrared-active transverse optical phonon modes, which are characterized by a maximum in the imaginary part of the medium’s permittivity. Here, in this Perspective article, we discuss an alternative strategy where the light pulses are instead tailored to match the frequency at which the real part of the medium’s permittivity goes to zero. This so-called epsilon-near-zero regime, popularly studied in the context of metamaterials, naturally emerges to some extent in all dielectric crystals in the infrared spectral range. We find that the light-matter interaction in the phononic epsilon-near-zero regime becomes strongly enhanced, yielding even the possibility of *permanently* switching both spin and polarization order parameters. We provide our perspective on how this hitherto-neglected yet fertile research area can be explored in future, with the aim to outline and highlight the exciting challenges and opportunities ahead.

## Introduction

The overwhelming majority of material systems are macroscopically characterized by a certain type of ordering, in the form for example of charge, magnetization, polarization and nematicity. Discovering how to therefore modify such order parameters within ultrafast timescales while incurring minimal losses represents not only a compelling and fundamental challenge in condensed matter physics but could also have profound technological implications, reflecting the fact that innumerable devices are rooted in the use of such orders and the switching thereof.

Ultrafast optical techniques, based on the use of femtosecond pulses of light, have provided a groundbreaking platform for both interrogating and stimulating ultrafast dynamics in condensed matter. The THz electric field intrinsic to ultrashort pulses can coherently couple to many different degrees of freedom. In the context of opto-spintronics, high-frequency vibrations of the crystal lattice, in particular, can have a significant impact on the orbital dynamics of the electrons, and through it, on spins^1–6^. The ultrafast excitation of phonons, resulting in a dramatic repopulation of the phononic system, is capable of modifying fundamental magnetic interactions^7,8^. Moreover, recent time-resolved X-ray scattering and electron diffraction experiments have demonstrated that the process of ultrafast demagnetization involves the incoherent transfer of angular momentum from spins to circularly-polarized phonons, on the time scale of femtoseconds. This has been appropriately dubbed the ultrafast Einstein-de-Haas effect^9,10^. By virtue of simple symmetry considerations, it is even feasible to realize the inverse process^11–13^, through which circularly-polarized vibrations of the lattice can give birth to magnetization on the same ultrashort time scale!

### Non-linear phononics and TO phonon modes

While the possibility of using lattice vibrations to affect macroscopic ordering has long been recognized, the research area of non-linear phononics – developed in large part by the research group led by A. Cavalleri (Max Planck Institute, Hamburg)—deserves special mention^14,15^. Non-linear phononics, broadly based on the microscopic mechanism of ionic Raman scattering^16–18^, uses mid- to far-infrared (IR) light pulses to resonantly drive IR-active transverse-optical (TO) phonon modes to large amplitude. The strong oscillations of the dipole moment couples non-linearly to Raman modes, resulting in the rectified modification of the equilibrium state. This method, inducing non-equilibrium atomic structures, has the power to temporarily create and control a wide variety of phases involving superconductivity^19,20^, ferroelectricity^21^ and magnetism^22^. Importantly, the optically-driven atomic motions can break crystal symmetries in manners that are entirely impossible under equilibrium conditions, with the dynamical deformations of the lattice being orders of magnitude stronger than anything that can be tolerated across longer timescales^22^. The nonlinear and coherent optical manipulation of the microscopic crystal structuring undeniably provides unique control over macroscopic ordering, not only in terms of the new otherwise-inconceivable functionalities that can be unlocked but also because of the ultrafast time scales in which they arise, often within femto- or pico-seconds.

So far, experimental works in the area of non-linear phononics have consistently hinged on the use of amplified laser systems based on difference-frequency generation (DFG)^23,24^. These light sources are highly convenient and practical for complex time-resolved experiments, particularly due to their table-top nature facilitating regular and uninterrupted use. They can offer broad tunability of the central wavelength *λ* in the mid-IR spectral range, pulse durations shorter than 100 fs, and electric field amplitudes exceeding megavolts per centimeter. In this regard, they straightforwardly enable non-linear phononics to be studied in a time- and color-resolved manner with a high degree of sensitivity and versatility. An important caveat that must be mentioned here is the spectral width Δ*λ* of the laser pulses, which is typically large (Δ*λ*/*λ* ~ 10%) due to the short pulse-durations necessary for DFG.

### Ultra-narrowband excitations targeting LO phonon modes

**Fig. 1:**
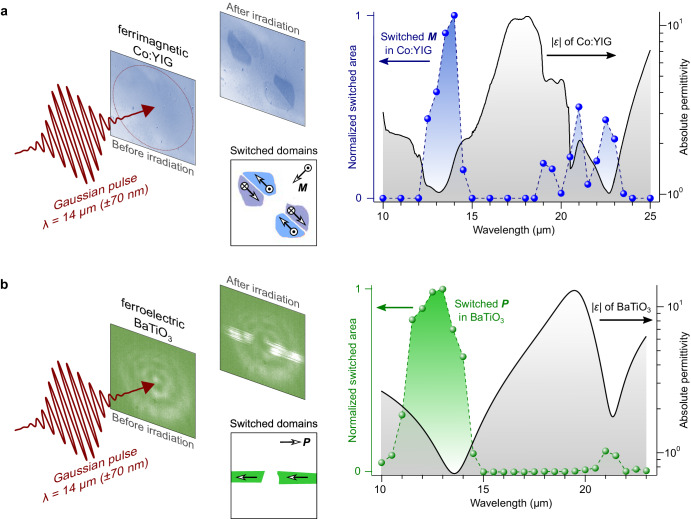
Switching of spontaneous order parameters in the epsilon-near-zero regime. Left: Schematic sketch of how a narrowband infrared pulse creates a permanent pattern of (**a**) switched magnetization (***M***) in ferrimagnetic cobalt-doped yttrium-iron-garnet (Co:YIG), and (**b**) switched polarization (***P***) in the ferroelectric barium titanate (BaTiO_3_). Right: Spectral dependence of the switching (points) and the absolute relative permittivity (*ε*) characteristic of the irradiated material (black line, plotted on a logarithmic scale). Adapted from refs. ^26,30^.

Recently, a series of experimental works have explored how spontaneous order parameters characteristic of various materials can be affected by IR pulses tuned to different spectral lines. The latter, delivered by the free-electron laser facility FELIX in the Netherlands^25^, have an extremely small spectral width, with Δ*λ*/*λ* being as small as 0.3%. Intriguingly, such ultra-narrowband IR pulses consistently seem to have the strongest effect when tuned to match not the frequencies of the TO phonon modes, as conventionally targeted in non-linear phononics, but rather the frequencies of the longitudinal optical (LO) phonon modes. For example, upon irradiating dielectric cobalt-doped yttrium-iron-garnet films with mid- and far-IR optical pulses (of amplitude ~40 kV/cm), the spontaneous magnetization can become permanently switched, as shown in Fig. 1a^26^. The unusual inhomogeneous spatial distribution of the switched magnetic domains can be explained in terms of the symmetries of the magnetoelastic interaction, as discussed qualitatively in ref. ^27^ and confirmed by micromagnetic calculations reported in ref. ^26^. Crucially, the spectrally-dependent amplitude of this switching (assessed through the size of the switched domains) scales qualitatively well with the population of LO phonon modes. Note that this pattern of magnetic switching – appearing within a timescale of ≈60 ps^28^—is entirely independent of the pump pulse’s polarization, and remains unaffected by subsequent impinging pulses. Alternatively, upon illuminating type-II antiferromagnetic nickel oxide with similar IR pulses, one can observe that the antiferromagnetic domain walls are most displaced when the wavelength *λ* of the impinging light again coincides with the frequency of the LO phonon modes^29^. Similar results are found even when irradiating the polar ferroelectric barium titanate (BaTiO_3_)^30^. Specifically, when exposing BaTiO_3_ to pulses tuned in frequency to match its characteristic LO phonons, both the 90° and 180° ferroelectric domains can become permanently switched (Fig. 1b). Again, in opposition to the Gaussian spatial distribution of the incident IR pulse, the switched domains are spatially inhomogeneous, which can be understood in terms of the symmetries of the piezoelectric displacement field. While experimentally unexplored as of yet, beam shaping should be able to modify the spatial distribution of both the switched magnetization and polarization.

It is therefore clear that the LO phonon modes appear to play a central role in the switching experiments, in stark contrast to the TO phonon modes that are conventionally excited in non-linear phononics. We emphasize the fact that ferrimagnetic iron-garnets, antiferromagnetic nickel oxide and ferroelectric BaTiO_3_ have very dissimilar crystal symmetries, causing the phonon modes in all these systems to have very different characters. Moreover, the spatial pattern of the switched magnetization and polarization follows not the microscopic electric fields but rather the macroscopic pulse shape. Perhaps most perplexing, the three aforementioned materials were all optically irradiated by transversely-polarized IR pulses coming at normal incidence. The LO phonon modes, being equivalent to longitudinal electromagnetic waves, therefore cannot even be directly excited to begin with. How then is it possible for the IR light-wave to interact with the LO phonon mode?

### The epsilon-near-zero regime

To attempt to elucidate the origin of this pronounced effect, Kwaaitaal et al. proposed that the common denominator in all these cases happens to lie in terms of the optical properties of the samples^30^, rather than in terms of its optical phonon modes. Let us rationalize this hypothesis by briefly revisiting the theory of optical phonons^31–33^. For a primitive unit cell containing *p* atoms, there are *n* = 3*p*-3 optical phonon modes. By virtue of the long-range Coulomb interaction, these optical phonons are split into LO and TO modes with characteristic frequencies *ω*_LO_ and *ω*_TO_ respectively. In polar solids that are cubic and diatomic, the LO-TO splitting is given by the Lyddane–Sachs–Teller relation^34^

1 $$\frac{{\omega }_{LO}^{2}}{{\omega }_{TO}^{2}}=\frac{{\varepsilon }_{0}}{{\varepsilon }_{\infty }},$$

where ${\varepsilon }_{0}$ and ${\varepsilon }_{\infty }$ are the static and high-frequency dielectric constants respectively. The LO-TO splitting often assures that *ω*_LO_ is well separated from *ω*_TO_, giving rise to the signature Restrahlen band (strong to total optical reflection) at frequencies *ω*_LO_ < *ω* < *ω*_TO_. In general, the *n* polar-optical phonon modes characteristic of a crystal can be treated as damped harmonic oscillators, leading to the Lowndes model of the permittivity^35^

2 $$\varepsilon (\omega )={\varepsilon }_{\infty }{\mathop{\prod }\limits_{i=1}^{n}}\frac{{\omega }_{{LO,i}^{2}}-{\omega }^{2}-i\omega {\gamma }_{LO,i}}{{\omega }_{{TO,i}^{2}}-{\omega }^{2}-i\omega {\gamma }_{TO,i}}$$

where ${\omega }_{{LO},i}$ and ${\omega }_{{TO},i}$ (${\gamma }_{{LO},i}$ and ${\gamma }_{{TO},i}$) are the frequencies (broadening) of the *i*-th LO and TO phonon modes, respectively.

**Fig. 2:**
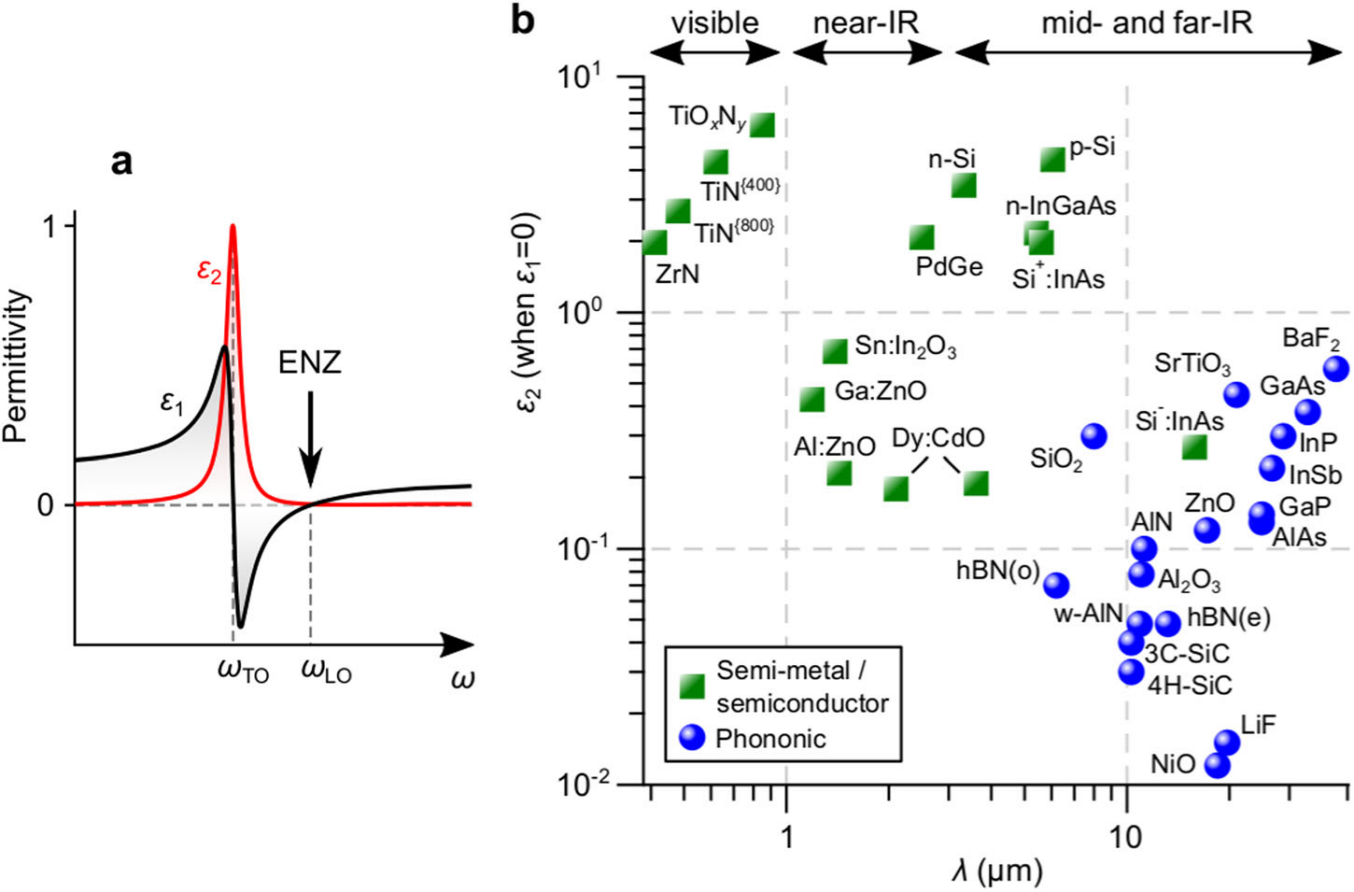
The correlation between optical phonon modes, permittivity and the ENZ regime. **a** Sketch of the typical frequency dependence of the complex permittivity *ε* = *ε*_1_ + i*ε*_2_ in the infrared (IR) spectral range. **b** Survey of typical values of *ε*_2_, evaluated at the point where *ε*_1_ = 0, identified in TiO_*x*_N_*y*_^60^, TiN (annealed at 400 °C and 800 °C)^61^, ZrN^62^, n- and p-doped Si^63^, n-doped InGaAs^64^, heavily-doped Si^+^:InAs^65^, PdGe^66^, Sn:In_2_O_3_^62^, Ga:ZnO^62^, Al:ZnO^62^, Dy:CdO^67^, SiO_2_^68^, BaF_2_^42^, SrTiO_3_^69^, GaAs^70^, lightly-doped Si^-^:InAs^65^, InP^71^, InSb^72^, GaP^73^, AlAs^74^, ZnO^75^, AlN^76^, Al_2_O_3_^77^, w-AlN^78^, hexagonal boron nitride (hBN) along the ordinary (o) and extraordinary (e) axis^79^, 3C-SiC^80^, 4H-SiC^81^, LiF^82^ and NiO^82^.

The complex frequency-dependent permittivity *ε*(*ω*) = *ε*_1_(*ω*) + i*ε*_2_(*ω*) fully describes the light-matter interaction, where *ε*_1_ accounts for the charge screening and *ε*_2_ the optical losses. TO phonon modes are identified by maxima in *ε*_2_ whereas LO phonon modes are marked by poles in the dielectric loss function Im(-*ε*^−1^), i.e., when *ε*_2_ / (*ε*_1_^2^ + *ε*_2_^2^) is maximized or, conversely, when (*ε*_1_^2^ + *ε*_2_^2^) / *ε*_2_ → 0. The latter is realized when *ε*_1_ crosses zero and *ε*_2_ is sufficiently small. This condition can be straightforwardly understood in terms of a simple Lorentzian oscillator model (Fig. 2a), where there is always a frequency at which *ε*_1_ goes from negative while crossing zero. Moreover, at this frequency, one often finds a low value of *ε*_2_ since the LO-TO splitting has naturally shifted the frequency *ω*_TO_ of the TO phonon mode - marked by maxima in *ε*_2_ - away from that of the LO one *ω*_LO_. The spectral position of the LO phonon mode, therefore, corresponds exactly to the wavelength at which the material’s permittivity goes to zero. Notice that, in the experiments described in Fig. 1, it was consistently observed that ultra-narrowband IR pulses - with Δ*λ*/*λ* being as small as 0.3% - had the largest impact on the magnetization or polarization when the entire bandwidth of the optical excitation fitted the point at which the material permittivity is minimized and approaches zero. If the frequency of the IR pulse was instead tuned to match that of the strongly-absorbing TO phonon modes, substantial heat-driven effects were observed, leading to effects including demagnetization^26^, strong birefringence^30^, or even irreversible structural damage of the sample^26^.

At the frequency at which a material’s permittivity approaches zero, i.e., in the so-called epsilon-near-zero (ENZ) regime^36–38^, the attributes of light-matter interaction become highly counter-intuitive. To illustrate this, let us consider the scenario of light being incident on a non-magnetized material under ENZ conditions. Snell’s law implies that the impinging light wave will only penetrate the ENZ material when close to normal incidence^39^. Moreover, continuity of the normal electric displacement field at the surface of the ENZ material implies that, if *ε* → 0, the electric field within the material must become strongly enhanced. This gives rise to intrinsically strong light-matter interaction, producing for example extraordinary levels of second and third harmonic generation^40^. Upon entering the material, the light-wave – with a group velocity ${v}_{g}=c\sqrt{\varepsilon }$ where *c* is the speed of light in vacuum^41^ – becomes dramatically “slow”, drastically increasing the interaction time of the light with the irradiated medium. Reciprocally, the phase velocity ${v}_{{ph}}=c/\sqrt{\varepsilon }$ and wavelength of the light-wave diverges, causing its electric field to become spatially uniform.

ENZ regimes can be found in a variety of materials in very different spectral ranges. The real part of the permittivity of gold and silver, for example, crosses zero in the ultraviolet spectral range^42^. However, such noble metals typically have substantial losses, i.e., large values of *ε*_2_. In the near-IR spectral range, transparent conducting oxides also feature zero-crossings of *ε*_1_, physically emerging due to plasmonic effects^43^. ENZ regimes can also be artificially constructed within so-called “metamaterials”^44,45^, most commonly comprising a sub-wavelength structuring of metallic and dielectric elements (in which *ε*_1_ < 0 and *ε*_1_ > 0, respectively)^46–48^. This combination allows one to study the ENZ regime in both metals and semiconductors. Based on its ability to make the light’s electric field slower, more in-phase and amplified, the ENZ regime has been shown to facilitate unusual effects including but not limited to optical cloaking^46^, photon tunneling^49^, negative refraction^50^, super-coupling^51^, control of emission^52^, levitation^53^ and colossal nonlinear interactions^54,55^. The latter nominally provide a basis for ultrafast and efficient optical switches^54^. However, such systems are somewhat lossy, with *ε*_2_ not decreasing much below unity in the visible to near-IR spectral ranges^36^. In contrast, by virtue of the intrinsic LO-TO splitting of phonon modes, dielectric materials hold the distinct advantage of having much smaller losses at ENZ points compared to those found in both semi-metals and semiconductors, as shown in Fig. 2b. As such, one can contend that such phononic materials provide the best possible realization of the ENZ regime.

What can one gain from the ENZ regime in the mid- to far-IR spectral ranges? Using the mechanism of nonlinear phononics, recent experiments in ferroelectrics have shown ultrashort transient effects on the ferroelectric polarization, without any permanent switching^56^. In contrast, it appears that excitations at the phononic ENZ conditions actually have the ability to drive a complete and permanent reversal of both polarization and magnetization^26–30^. The significance of the ENZ regime is strongly underscored by the finding that the switching of polarization in BaTiO_3_ experiences a redshift on the order of ~400 nm (<3% of *λ*) when rotating the linear polarization of the incident IR pulse by 90°^30^. This redshift follows exactly that of the permittivity and ENZ point of BaTiO_3_, which are anisotropic within the plane of the crystal^57^. The fact that ENZ conditions emerge organically from the solid’s ionic lattice suggests that the demonstrated mechanism of reversal could even be universal, capable of enhancing light-matter interaction and even permanently switching order parameters in a wide variety of systems. We envision this regime could be particularly fruitful when considered in multiferroics^58^, since the same excitation could be capable of simultaneously affecting different order parameters.

In our view, despite a myriad of experimental works utilizing THz and IR pulses, the phononic ENZ regime has remained under-investigated primarily because of the difficulty associated with obtaining ultra-narrowband IR pulses. Now, with the possibility of using both table-top systems and free-electron lasers to obtain such IR light pulses^23,59^, we are technologically capable of exploring this area. Free-electron lasers can deliver transform-limited pulses with bandwidth as small as 0.3%, allowing the entire bandwidth of the pulse to ideally satisfy the ENZ condition. Table-top laser systems, while hugely convenient for experiments, usually generate IR pulses that are rather broadband and are thus poorly suited for selectively targeting phononic ENZ points. While one can simply use frequency filters to reduce the bandwidth of initially broadband pulses, the spectral narrowing sacrifices a significant amount of pulse energy, making it completely insufficient for such experiments targeting the ENZ regime. Moreover, this approach does not conveniently facilitate tuning of the pulse’s central wavelength. Nevertheless, it is our hope that advances in laser technologies will eventually provide table-top systems that can deliver highly-narrowband wavelength-tunable IR pulses while preserving high pulse energy. Encouraging progress in this direction has already been reported by Cartella et al. in ref. ^23^, in which tabletop chirped-pulse DFG produced mid-IR pulses with relative bandwidth down to 1.6%. In the meantime, we must note that there are more than ten free-electron laser facilities worldwide that can supply the ultra-narrowband IR pulses prerequisite for studies of the phononic ENZ regime in dielectric materials. We thus hope that this perspective inspires other experimentalists to explore this rich and fertile research area, which certainly has excellent potential to present a new paradigm for future opto-spintronics.
